# Maternal Age at Menarche Gene Polymorphisms Are Associated with Offspring Birth Weight

**DOI:** 10.3390/life13071525

**Published:** 2023-07-07

**Authors:** Yuliya Reshetnikova, Maria Churnosova, Vadim Stepanov, Anna Bocharova, Victoria Serebrova, Ekaterina Trifonova, Irina Ponomarenko, Inna Sorokina, Olga Efremova, Valentina Orlova, Irina Batlutskaya, Marina Ponomarenko, Vladimir Churnosov, Natalya Eliseeva, Inna Aristova, Alexey Polonikov, Evgeny Reshetnikov, Mikhail Churnosov

**Affiliations:** 1 Department of Medical Biological Disciplines, Belgorod State National Research University, 308015 Belgorod, Russia; 130226@bsu.edu.ru (Y.R.); churnosovamary@gmail.com (M.C.); ponomarenko_i@bsu.edu.ru (I.P.); sorokina@bsu.edu.ru (I.S.); efremova@bsu.edu.ru (O.E.); orlova@bsu.edu.ru (V.O.); bat@bsu.edu.ru (I.B.); 1256888@bsu.edu.ru (M.P.); churnosov_v@bsu.edu.ru (V.C.); eliseevanb78@mail.ru (N.E.); aristova@bsu.edu.ru (I.A.); polonikov@rambler.ru (A.P.); reshetnikov@bsu.edu.ru (E.R.); 2 Research Institute for Medical Genetics, Tomsk National Research Medical Center of the Russian Academy of Sciences, 634050 Tomsk, Russia; vadim.stepanov@medgenetics.ru (V.S.); anna.bocharova@medgenetics.ru (A.B.); vika.serebrova@medgenetics.ru (V.S.); ekaterina.trifonova@medgenetics.ru (E.T.); 3 Department of Biology, Medical Genetics and Ecology and Research Institute for Genetic and Molecular Epidemiology, Kursk State Medical University, 305041 Kursk, Russia

**Keywords:** birth weight, single nucleotide polymorphism, association, age of menarche

## Abstract

In this study, the association between maternal age at menarche (AAM)-related polymorphisms and offspring birth weight (BW) was studied. The work was performed on a sample of 716 pregnant women and their newborns. All pregnant women underwent genotyping of 50 SNPs of AAM candidate genes. Regression methods (linear and Model-Based Multifactor Dimensionality Reduction (MB-MDR)) with permutation procedures (the indicator p_perm_ was calculated) were used to identify the correlation between SNPs and newborn weight (transformed BW values were analyzed) and in silico bioinformatic examination was applied to assess the intended functionality of BW-associated loci. Four AAM-related genetic variants were BW-associated including genes such as *POMC* (rs7589318) (β_additive_ = 0.202/p_perm_ = 0.015), *KDM3B* (rs757647) (β_recessive_ = 0.323/p_perm_ = 0.005), *INHBA* (rs1079866) (β_additive_ = 0.110/p_perm_ = 0.014) and *NKX2-1* (rs999460) (β*_recessive_* = −0.176/p_perm_ = 0.015). Ten BW-significant models of interSNPs interactions (p_perm_ ≤ 0.001) were identified for 20 polymorphisms. SNPs rs7538038 *KISS1*, rs713586 *RBJ*, rs12324955 *FTO* and rs713586 *RBJ*–rs12324955 *FTO* two-locus interaction were included in the largest number of BW-associated models (30% models each). BW-associated AAM-linked 22 SNPs and 350 proxy loci were functionally related to 49 genes relevant to pathways such as the hormone biosynthesis/process and female/male gonad development. In conclusion, maternal AMM-related genes polymorphism is associated with the offspring BW.

## 1. Introduction

BW is one of the key indices of a newborn’s conditions/pregnancy outcome and has meaningful prognostic value as a biological predictor of future health [1,2,3]. Newborns with sufficiently high/low BW values are characterized by a considerably raised risk of health disorders (including mortality) in their life-course in comparison with newborns with average BW values [1,2,3,4]. BW has strong phenotypic/genetic correlations of reverse orientation with the risk of later-life cardiometabolic phenotypes/disorders such as coronary artery disease, systolic blood pressure, type 2 diabetes mellitus/glycemic traits and positive phenotypic/genetic correlations with anthropometric/obesity-related traits such as BMI, height, waist circumference, etc. [1,2,3,5,6,7,8,9].

BW is related to the influence of both maternal and fetal genetic factors and also environment contributions [10,11,12,13]. The available modern scientific data demonstrating the contribution of the above groups of factors to BW are very variable: fetal genetic factors can explain 24–69% of the BW variance, maternal genetic factors—3–22% and environmental influences—20–30% [10,11,12]. Attention is drawn to the expressive variability of the estimates of the contribution of maternal (more than seven times) and fetal (about three times) genomes to BW available in the literature, which may indicate the presence of complex population-specific, incompletely understood and studied effects of maternal/fetal genomes and their influence on BW with significant modifying effects of environmental factors. In one of the latest genome-wide associative studies (GWAS) focused on the influence of the maternal genome on BW, it was shown that seven out of ten GWAS-significant for BW loci showed the effects of the maternal genome on BW through the intrauterine environment [13]. These genetic determinants were linked with mothers’ phenotypes such as fasting glucose levels (*TCF7L2*; *GCC*; *MTNR1B*), levels of sex hormone (*CYP3A7*) and pregnancy duration (*EBF1*) [13].

Importantly, on the one hand, the above data indicate the importance of the maternal genome, which makes a significant contribution to BW (it determines both the effects of common alleles in the fetus and the intrauterine environment effects of the maternal genotype [13,14]). On the other hand, the influence of common genetic variations of the mother on BW has been poorly studied [13]. Moreover, it is the data on specific genetic factors of the maternal organism that strongly correlates with BW that may be of interest as promising biomarkers for use in practical obstetrics for prognostic purposes (identifying risk groups for the birth of high/low-weight fetuses) [13,14,15].

Among the various key woman phenotypes (BMI, the level of sex hormones, etc.), the age of menarche (AAM) is of paramount importance [16,17]. AAM is a comprehensive pointer of the woman hypothalamic-pituitary-ovarian system functioning and the most important biological highlighter of a woman’s fertility [17]. AAM is largely determined by the genetic component (57–82% of AAM variability is associated with heredity) [18,19,20]. Early/late AAM is linked with the risk of various health-related disorders in adulthood such as obesity, impaired glucose metabolism and insulin resistance, metabolic syndrome, diabetes, cardiovascular diseases, etc. [16,21]. AAM genes are associated with BMI-related traits (earlier AMM causes higher adult BMI) [22,23,24,25,26,27], various female benign (endometriosis/leiomyoma/endometrial hyperplasia) [28,29,30] and malignant (breast/endometrial cancers) [31] reproductive system disorders and pregnancy complications (gestational diabetes mellitus) [32]. These maternal sex-steroid-sensitive and glucose-involved pathways may be potentially BW-significant [13].

There are very few studies aimed at finding associations between maternal AAM and offspring BW [33,34,35,36]. Wang et al. on a sample of 17,571 subjects from China described associations of early maternal AAM with increased BMI of offspring [33]. It was shown that the children of mothers with early AAM were taller at the age of 1 year and had a higher BMI at the ages of 7–8 years [34]. Similarly, the children of mothers with early AAM were distinguished by taller height, greater weight, BMI and fat mass index at 9 years old [35]. It is believed that early menarche correlates with increased pubertal growth and causes a high adolescent/adult BMI [25,26,27] and may lead to the birth of offspring with increased BW due to this [15]. The genetic links of early menarche with an increased risk of gestational diabetes are shown [32], which also leads to the birth of large-BW newborns. Thus, the available convincing literature data allow one to assume a link between early maternal AAM and increased BW of offspring. At the same time, there is evidence of an association between early maternal AAM with preterm birth [37] and suggestions are made about its connection with low BW [38].

It should be noted that at the moment there are no specific genetic data on the association of AAM-related polymorphism with BW (both according to GWAS data and other associative studies). It has been suggested that the intergenerational correlations between maternal AAM and offspring BW (in particular, BMI in early puberty) may be based on some common genetic mechanisms (e.g., *LIN28B*, *PXMP3* genes) [33,39], but the specific genetic factors of these relationships are still unknown [33]. However, currently, there is no doubt about the proven scientific facts of genetic correlations of numerous maternal menarche-related traits such as height, BMI, glucose level, systolic blood pressure, etc. with BW [3,13,15]. In addition, low BW was correlated with early AAM [21,40] and causal genetic effect of BW on AAM (lower BW determined increased risk of earlier AAM) [41,42] and AAM-linked phenotypes in adults such as obesity-related (BMI; height; waist circumference [positive genetic correlations]) and cardiometabolic (coronary artery disease; systolic blood pressure; type 2 diabetes mellitus [inverse genetic correlations]) traits [1] were found. However, at the same time, the role of maternal AAM candidate genes in the formation of BW remains practically unexplored. This study is aimed at filling this gap. Its purpose was to investigate the associations of maternal AAM genes polymorphism with offspring BW.

## 2. Materials and Methods

### 2.1. Study Subjects

**Figure 1:**
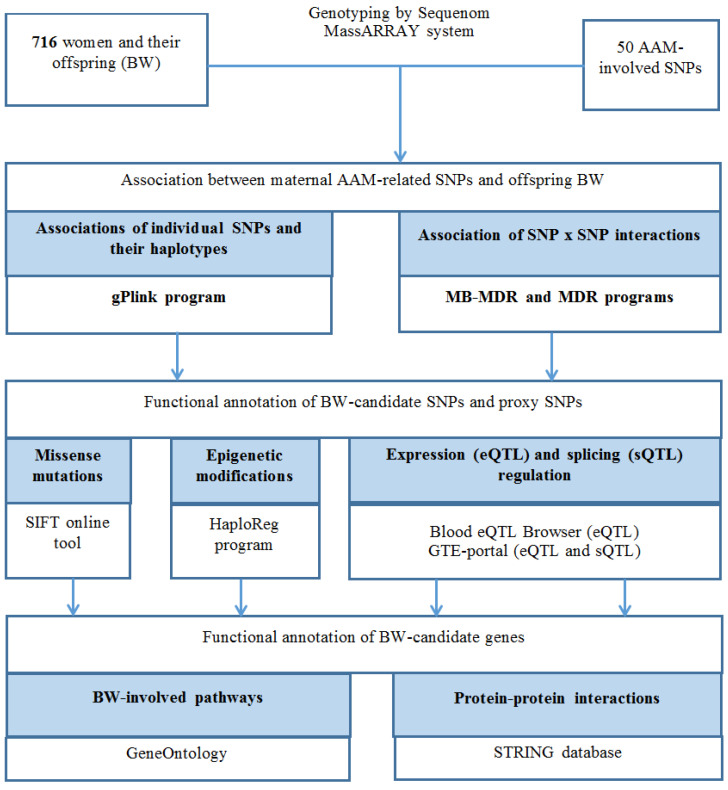
Study design.

The study design outline is presented in Figure 1. The participants of our study were 716 women recruited at the Belgorod Regional Clinical Hospital (Perinatal Center) in the period 2008–2017. All methods/procedures of this study were approved by the Ethics Council of Belgorod State University. Signed informed consent from the participants was a prerequisite for inclusion in the study. All subjects of the current study participated in previously conducted clinical and genetic studies of pregnancy outcomes/disorders (preeclampsia, fetal growth restriction) (detailed biomedical, anamnestic information, data on the course of a real pregnancy and its outcomes were collected about each subject; BW parameter was taken from the medical record of childbirth) [43,44,45,46,47,48,49,50]. The group of subjects included women who met the following criteria: singleton pregnancy (24–41 weeks of gestation) ending in a live birth, Russian origin [self-reported], born in the Central region of Russia [51,52]. Women with parameters such as age younger than 16, multiple pregnancies, anomalies of the uterus/cord/fetus, childbirth <24 weeks of pregnancy did not participate in the study (exclusion criteria). The detailed characteristics of the sample are presented in Table 1.

### 2.2. DNA Extraction, AMM-Involved SNPs Selection, Genotypes Testing

The maternal venous blood (taken from the cubital vein) was exploited to gain DNA (the phenol/chloroform/ethanol DNA extraction/purification method has been described in detail in [53]). The quality/quantity of extracted DNA was evaluated on a NanoDrop2000 micro volume spectrophotometer and then DNA samples were stored in a kelvinator at −80 °C.

When selecting polymorphisms for this genetic study, special criteria were taken into account [24,28,29,30] such as; (1) association with AAM and/or phenotypes that share pathways with AAM (weight/height/BMI/obesity/vitamin D metabolism, etc.) (Supplementary Table S1); (2) functionality (epigenetic role, association with gene expression etc.); (3) the frequency of the polymorphic variant is not less than 5%. Using these criteria, the literature data on this issue (presented in detail in Supplementary Table S1) and the materials of our previous studies [24,28,29,30], a total of 50 loci of AAM candidate genes were included in the study. All these loci had important functional significance (Supplementary Table S2) (according to the information provided in the HaploReg database [54]). Among the 50 loci included in the study, 13 SNPs were associated with AAM according to GWAS data and 26 SNPs were AAM-link according to the results of other associative studies (Supplementary Table S1); 15 SNPs were associated with various an anthropometric parameters (Supplementary Table S1); 10 SNPs out 50 loci (such as rs4633, *COMT*; rs1544410, *VDR*; rs2252673, *INSR*; rs222003, rs222020, *GC*; rs7766109 *F13A1*; rs1884051, rs3020394 *ESR1*, rs3756261 and *EGF*; rs12324955 *FTO*) were not directly related to AAM, but they correlated with AAM-significant phenotypes/disorders [e.g., metabolism of vitamin D; polycystic ovary syndrome, etc. (Supplementary Table S1)] and had significant opportunity functionality (Supplementary Table S2). Importantly, we previously used this SNP panel in conducting associative studies of AAM and anthropometric parameters (height/BMI) of women [24], proliferative uterine diseases (fibroids/endometriosis/endometrial hyperplasia) [28,29,30] in the studied population (Central Russia), which allows us to conduct a correct comparative analysis of the role of BW-associated polymorphisms in the formation of other phenotypes/diseases of the female reproductive system in this work.

The DNA SNPs were identified by Sequenom MassARRAY system (“Seqeunom”, San Diego, CA, USA) in Tomsk National Research Medical Center of the Russian Academy of Sciences (Tomsk, Russia). Quality control of SNPs genotyping was accomplished based on the following indicators: call rate (>90%); success rate of duplicate check (>99%); success rate of the blank check (>90%) [30,55]. In total, 49 out of 50 analyzed loci passed the quality control procedure (for rs11724758 *FABP2*, the call rate was 84.25%, so it was excluded from the subsequent analysis).

### 2.3. SNPs Association Analysis

For all studied AAM-involved SNPs, the compliance of their distribution (alleles/genotypes) with the Hardy–Weinberg law was checked [56,57]. The link of BW with polymorphisms and their haplotypes was evaluated in the gPlink (Java-tied versia 2.050) program [58] by linear regression (three genetic models [additive; dominant; recessive] [59] were considered). Methods and programs of the same name MDR [60,61] and MB-MDR [24,62] were applied to modeling BW-significant interlocus interactions. For genetic computations, transformed BW values were used (real BW values with non-normal distribution were reduced to a normal distribution by QQ-plot function in the R program [24]). The calculations used covariates (age at menarche, pre-pregnancy BMI, presence in the anamnesis of hypertension and fetal growth restriction, presence of current pregnancy complications (fetal growth restriction, preeclampsia and a combination of fetal growth restriction with preeclampsia; date from Table 1) (covariates were included as independent variables in the regression analyses) and procedures that minimize the risk of false positive results (permutation test was applied [63,64]). Importantly, when selecting BW-significant interlocus interaction models for permutation procedures, we introduced additional Bonferroni corrections (the quantity of possible combinations of 49 SNPs was taken into account) and as a result, values of the significance level such as *p* < 4 × 10^−5^ (<0.05/1176; two-locus models), *p* < 3 × 10^−6^ (<0.05/18424; three-locus models) and *p* < 2 × 10^−7^ (<0.05/211876; four-locus models) were set as the “threshold”. Indicators with p_perm_ < 0.017 (for individual SNPs; an additional Bonferroni correction was introduced, equal to 3 according to the number of calculated genetic models) and p_perm_ ≤ 0.001 (for models of interlocus interactions) were taken as threshold values when assessing the statistical significance of the established associations. In our sample of subjects (*n* = 716), with a planned study capacity of at least 80%, it is possible to detect differences in BW at the level of 86–142 g (additive model), 138–156 g (dominant model), 146–623 g (recessive model). Statistical power for BW-associated SNPs was calculated by Quanto 1.2.4 [65].

### 2.4. BW-Involved SNPs/Genes Potential Functions

For BW-associated loci and proxy polymorphisms (r^2^ is 0.8 or more [66,67]), we evaluated the potential functionality that may underlie their association with BW [68,69,70]. To solve this objective, we used the widely utilized in silico methodology [71,72,73,74] and the following six modern bioinformatic tools (1) Blood eQTL resource [75], (2) STRING [76], (3) HaploReg [54], (4) SIFT [77], (5) GeneOntology [78] and (6) GTE [79].

## 3. Results

### 3.1. Study Participants’ Characteristics

The study group included women aged 16–45 years, with a single pregnancy ending in a live birth, Russian origin, born in the Central region of Russia. The main characteristics of the women/newborn subjects included in our study (*n* = 716) are presented in Table 1. As follows from Table 1, a lower BW has been associated with maternal parameters such as late menarche (*p* = 0.02), low pre-pregnancy BMI (*p* < 0.0001), a history of hypertension (*p* = 0.0002) and fetal growth restriction (*p* < 0.0001), as well as with complications of the current pregnancy (fetal growth restriction and a combination of fetal growth restriction with preeclampsia (*p* = 0.0001)). The above phenotypic characteristics were covariates when evaluating the association of AAM-involved SNPs with BW.

### 3.2. SNPs/Haplotypes Association Analysis

The allele/genotypic distribution of the considered AAM-involved 49 SNPs was in full respect of the Hardy–Weinberg law (Supplementary Table S3) (the analysis of the correspondence of the observed distribution of genotypes to the expected distribution according to the Hardy–Weinberg law can serve as one of the indicators of the quality of the performed genotyping of SNPs; if the expected/observed distribution of genotypes is appropriate, the quality of the experimental studies performed can be considered satisfactory and the results of genotyping can be used in the analysis of associations). Four SNPs out of 49 analyzed loci were BW-associated, such as rs7589318 *POMC* (allele A [additive model] β = 0.202 *p* = 0.015 p_perm_ = 0.015 [power 81.59%]), rs757647 *KDM3B* (allele T [recessive model] β = 0.323 *p* = 0.004 p_perm_ = 0.005 [power 94.88%]), rs1079866 *INHBA* (allele C [additive model] β = 0.110 *p* = 0.014 p_perm_ = 0.014 [power 79.07%]) and rs999460 *NKX2-1* (allele A [recessive model] β = −0.176 *p* = 0.014 p_perm_ = 0.015 [power 80.21%]) (Table 2). The link of haplotypes with BW was not confirmed by the results of permutation testing (p_perm_ > 0.05) (Supplementary Table S4).

### 3.3. BW-Involved SNP × SNP Interactions

For 20 SNPs out 50 examined loci, we modeled ten BW-significant SNP-SNP interplay models (p_perm_ ≤ 0.001) (Table 3). Three loci such as rs7538038 *KISS1*, rs713586 *RBJ*, rs12324955 *FTO* and two-locus interaction rs713586 *RBJ*xrs12324955 *FTO* have been included in 30% of BW-significant models. The most appreciable exposure on BW (Wald value was maximum) was registered for a model consisting of four SNPs such as rs713586 *RBJ*, rs3020394 *ESR1*, rs1782507 *FSHB* and rs12324955 *FTO* (WH_BW_ = 56.83 [p_perm_ < 0.001]). The combinations of genotypes determining a higher BW have the greatest statistical significance (rs222003 GC *GC*xrs4986938 GA *ESR2*xrs2013573 GA *UGT2B4* [*beta_BW_* = 1.18 *p* = 0.0000003], rs999460 GG *NKX2-1*xrs12444979 CC *GPRC5B*xrs4374421 TT *LHCGR* [*beta_BW_* = 0.50 *p* = 0.000002]). The most perceptible phenotypic (BW) effect was also noted for the BW-enhancing combination of genetic variants (rs10441737 CC *ZNF483*xrs1073768 GA *GHRH*xrs7589318 GG *POMC*xrs4633 CC *COMT* [*beta_BW_* = 3.01 *p* = 0.00002]) (Supplemental Table S5).

**Figure 2:**
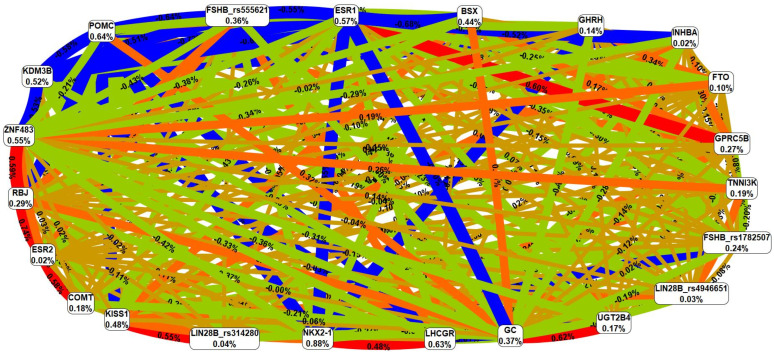
The entropy graph of gene × gene (SNP × SNP) interactions with birth weight. The figure outlines the gene × gene (SNP × SNP) interactions within the 2-, 3-, and 4-locus models obtained by the MB-MDR method. The percentage at the bottom of each gene (SNP) represents its entropy, and the percentage on each line represents the percentage of interaction between the 2 genes (SNPs). The red and orange lines indicate a stronger and weaker synergism, respectively, brown—an independent effect of individual gene (SNPs), green—weaker antagonism, blue—stronger antagonism.

Amid the BW-significant epistatic interplays (Figure 1), antagonistic liaisons (indicated in Figure 1 in blue) play a noteworthy role, in which the SNPs rs7538038 *KISS1*, rs7589318 *POMC*, rs999460 *NKX2-1* and rs3020394 *ESR1* occupy a central place (each of these SNPs has been involved in four–seven distinct paired antagonistic interoperations). The most visible independent contribution to BW was registered for rs999460 *NKX2-1* [0.88%] and rs7589318 *POMC* [0.64%] (Figure 2).

### 3.4. Functional of BW-Candidate SNPs

Using the in silico methodology, the potential functionality of 22 BW-associated loci and 350 proxy SNPs (their connections with missense mutations; epigenetic modifications; regulation of expression/splicing; common pathways) was evaluated. The in silico approach allows using modern bioinformatic tools (as a rule, they include previously obtained experimental results on the functional value of SNP/genes) to obtain data on the putative functional role of the studied SNP/genes in the body. SNPs associated with missense mutations lead to amino acid substitutions in the encoded polypeptide, which can determine the change/disruption of the function of this protein. Loci associated with epigenetic modifications can regulate the transcriptional activity of genes (by influencing the promoter/enhancer regions of the genome, interaction with transcription factors and regulatory proteins, etc.), which ultimately determines the amount of protein product of these genes. Polymorphisms affecting the regulation of gene expression and splicing determine the level of transcription of mRNA from a given gene and the level of its alternative splicing, which as a result will affect the quantitative representativeness of proteins in the body controlled by these genes.

*SNPs and missense mutations.* Four non-synonymous loci amid 350 proxy SNPs were founded such as rs11676272 *ADCY3* (Ser107Pro ADCY3; r^2^ with BW-associated rs713586 *RBJ* equal 0.98), rs61742688 *GPRC5B* (Asn268Lys GPRC5B; r^2^ = 0.92 with rs12444979 *GPRC5B*), rs4680 *COMT* (Val158Met COMT; r^2^ = 0.99 with rs4633 *COMT*) and rs4889 *KISS1* (Pro81Arg KISS1; r^2^ = 0.98 with rs7538038 *KISS1*). Two amino acid substitutions (Ser107Pro ADCY3 and Asn268Lys GPRC5B) have a SIFT rating of “BENIGN” and also two other amino acid substitutions (Val158Met COMT and Pro81Arg KISS1) were labeled as “DELETERIOUS”.

*SNPs and epigenetic modifications.* A total of 22 BW-causal loci and 350 proxy SNPs were studied in relation to their connection with various epigenetic modifications of DNA. The significant estimated regulatory potential was established for all 22 BW-associated loci and 316 proxy SNPs (out 350 proxy loci, 90.26%), among which the overwhelming number of genetic variants have been located in transcription factors(TF)-binding domains (313 SNPs out 372 loci, 84.14%), 93 SNPs (25.00%)—in enhancer regions, 40 SNPs (10.75%)—in promoter regions, 81 polymorphisms (21.77%)—in DNAase-hypersensitive sites, 32 SNPs (8.60%)—in areas of interaction DNA with regulatory proteins, 14 SNPs (3.76%)—in conservative human genome fields (Supplementary Table S6).

Importantly, all four BW-causal variants have appreciable purported regulatory actions, e.g., rs757647 *KDM3B* has been located at the DNAase-hypersensitive province in cultured trophoblast cells, in the enhancer-impact DNA positions in mesenchymal stem and adipocyte cells, fatty nuclei, placenta, amnion, ovaries, a genome binding site to MAFK (regulatory protein) and CTCF (TF) (Supplementary Table S2). More examples, SNP rs999460 *NKX2-1* defines the DNA responsiveness to five TFs (AIRE, Arid5a, Foxa, Pax-4, STAT), and locus rs1079866 *INHBA*—to two TFs (HNF4,YY1) (Supplementary Table S2).

In total, 22 BW-causal SNPs with 316 proxy SNPs have regulatory (epigenetic) effects on 25 genes (*ADCY3*; *BSX*; *C11orf46*; *COMT*; *REN*; *RBJ*; *EFR3B*; *ESR1*; *ZNF483*; *ESR2*; *GC*; *FSHB*; *GHRH*; *GPR139*; *GPRC5B*; *INHBA*; *IQCK*; *KDM3B*; *KISS1*; *LHCGR*; *LIN28B*; *NKX2-1*; *TNNI3K*; *FTO*; *UGT2B4*;) including in BW-significant maternal organs (ovaries, adipose tissue), provisional organs (placenta, amnion), fetal cell cultures (cultured trophoblast cells, ectoderm and mesoderm cells, mesenchymal stem cells, etc. (Supplementary Table S6).

*SNPs and expression(eQTL)/splicing(sQTL) regulation.* The Blood eQTL Browser information indicates that five BW-associated loci (rs1073768 *GHRH*; rs713586 *RBJ*; rs4633 *COMT*; rs7589318 *POMC*; rs10441737 *ZNF483*) (Supplementary Table S7) and eight high-linked SNPs with three BW-causal variants (rs713586 *RBJ*; rs10441737 *ZNF483*; rs4633 *COMT*) (Supplementary Table S8) were eQTL-important in the human blood for six genes (*CENPO*; *ADCY3*; *RBJ*; *KIAA0368*; *MANBAL*; *COMT*).

Based on GTE-portal data, Supplementary Tables S9 and S10 show SNPs hosted in expression regulation genome points (15 BW-causal loci (68.18%) and 225 proxy loci (64.29%) and determinate the *mRNA* level of 28 genes (*REN*; *ADCY3*; *ARL14EP*; *ARVCF*; *CENPO*; *COMT*; *RBJ*; *DNAJC27-AS1*; *DNMT3A*; *EFR3B*; *SYNE2*; *ESR2*; *FSHB*; *GPRC5B*; *HACE1*; *POMC*; *KIAA0368*; *KIF20A*; *KNOP1*; *LIN28B*; *LINC00577*; *LRRIQ3*; *MANBAL*; *MROH8*; *MTHFD1*; *REEP2*; *RP4-710M3.1*; *UGT2B4*) in several human (maternal) organism organs/tissues (Supplementary Tables S9 and S10) including BW-impact organs such as adipose (visceral/subcutaneous) (*DNAJC27-AS1*; *CENPO*; *KNOP1*; *ADCY3*; *ARL14EP*; *REEP2*; *GPRC5B*), ovary (*KNOP1*; *ARL14EP*), thyroid (*ARVCF*; *KNOP1*; *ARL14EP*; *LRRIQ3*; *RP4-710M3.1*), adrenal gland (*KNOP1*; *ARL14EP*), skeletal muscle (*COMT*; *FSHB*; *GPRC5B*) and whole blood (*CENPO*; *ADCY3*; *ARL14EP*; *RBJ*). Besides, 11 BW-causal loci (Supplementary Table S11) and 257 LD SNPs (Supplementary Table S12) have been identified as splicing regulators of 12 genes (*TXNRD2*; *DNAJC27-AS1*; *ADCY3*; *ARL14EP*; *ARVCF*; *CDC25C*; *FAM53C*; *KNOP1*; *LIN28B-AS1*; *COMT*; *SFTA3*; *ZBTB1*). The important sQTL role of BW-involved loci was registered by us for a diverse range of BW-significant tissues of the maternal organism, such as adipose (visceral/subcutaneous) (*KNOP1*; *ADCY3*; *ARL14EP*; *ARVCF*; *COMT*), skeletal muscle (*KNOP1*; *ARL14EP*; *COMT*), ovary (*KNOP1*; *COMT*), thyroid (*KNOP1*; *ADCY3*; *SFTA3*; *ARL14EP*; *COMT*), adrenal gland (*KNOP1*; *COMT*; *ARL14EP*) and whole blood (*ARL14EP*; *COMT*).

Interestingly, the BW-raising allele A rs7589318 *POMC* has been correlated with lower *RBJ mRNA* production in blood (*Z* = −4.94) (Supplementary Table S7) and higher splicing level of *ADCY3* in subcutaneous adipose (β = 0.35) and thyroid (β = 0.26) (Supplementary Table S11). BW-increasing allele T rs757647 *KDM3B* was linked with low expression *REEP2* in subcutaneous adipose (β = −0.25) (Supplementary Table S9) and higher *CDC25C* splicing value (β = 0.77) in EBV-transformed lymphocytes (Supplementary Table S11).

*BW-involved pathways.* A deep comprehensive analysis of the functionality of 22 BW-significant loci and 350 proxy SNPs, effectuated at previous stages of the study, showed the involvement of 49 genes in BW, including due to missense mutations (four genes such as *ADCY3*, *GPRC5B*, *COMT*, *KISS1*), epigenetic modifications (25 genes such as *ADCY3*, *BSX*, *C11orf46*, *COMT*, *EFR3B*, *ESR1*, *ESR2*, *FSHB*, *FTO*, *GC*, *GHRH*, *GPR139*, *GPRC5B*, *INHBA*, *IQCK*, *KDM3B*, *KISS1*, *LHCGR*, *LIN28B*, *RBJ*, *NKX2-1*, *REN*, *TNNI3K*, *UGT2B4*, *ZNF483*), eQTL (28 genes such as *ADCY3*, *ARL14EP*, *ARVCF*, *CENPO*, *COMT*, *RBJ*, *DNAJC27-AS1*, *DNMT3A*, *EFR3B*, *ESR2*, *FSHB*, *GPRC5B*, *HACE1*, *KIF20A*, *KIAA0368*, *KNOP1*, *LIN28B*, *LINC00577*, *LRRIQ3*, *MANBAL*, *MROH8*, *MTHFD1*, *POMC*, *REEP2*, *REN*, *RP4-710M3.1*, *SYNE2*, *UGT2B4*) and sQTL (12 such as *ADCY3*, *ARL14EP*, *ARVCF*, *CDC25C*, *COMT*, *DNAJC27-AS1*, *FAM53C*, *KNOP1*, *LIN28B-AS1*, *SFTA3*, *TXNRD2*, *ZBTB1*) regulation. A detailed analysis of the 49 BW-linked genes’ biological pathways, conducted using nine different databases integrated into the Gene Ontology resource (released 2023-03-06), revealed more than 10 different pathways (Supplementary Table S13), among which hormone/gonad-related pathways prevail: peptide hormone biosynthesis (R-HSA-209952; Fold Enrichment (FE) parameter > 100; p-_FDR_ = 0.011), regulation of endocrine process (GO:0044060; FE = 44.76; p-_FDR_ = 0.007), ovulation cycle process (GO:0022602; FE = 43.04; p-_FDR_ = 0.003), ovarian follicle development (GO:0001541; FE = 33.78; p-_FDR_ = 0.015), male gonad development (GO:0008584; FE = 18.52; p-_FDR_ = 0.005) and regulation of hormone levels (GO:0010817; FE = 7.45; p-_FDR_ = 0.006).

**Figure 3:**
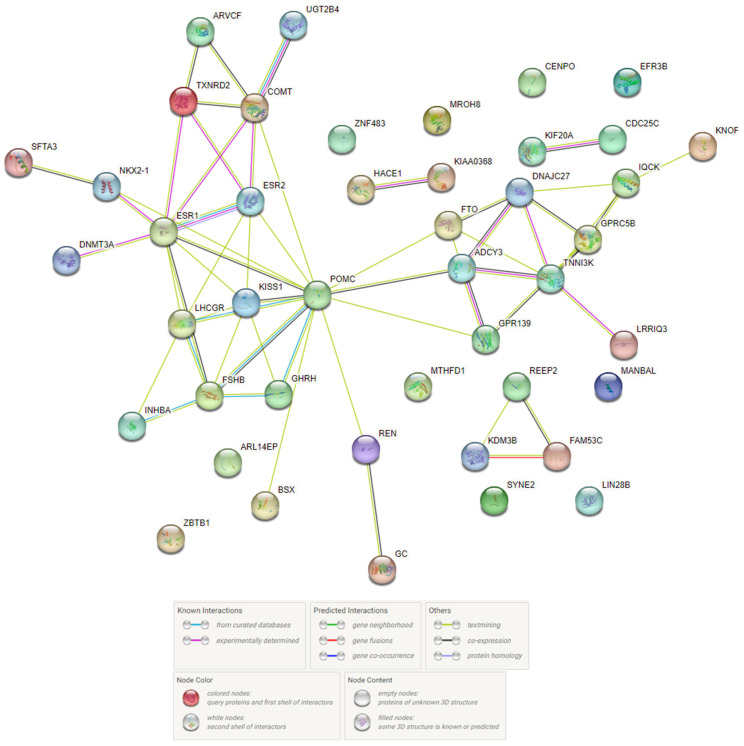
BW-involved protein-protein interaction networks inferred using the STRING resource.

**Figure 4:**
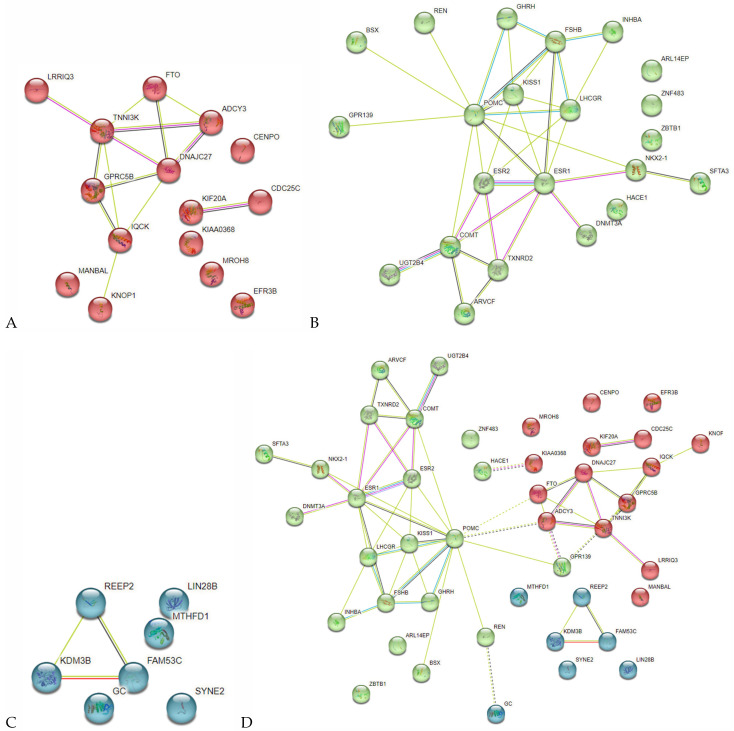
BW-involved protein-protein interaction clusters inferred using the STRING resource (three groups of P-Pi clusters are highlighted in color: cluster 1, red (**A**); cluster 2, green (**B**); cluster 3, blue (**C**); summary three clusters (**D**)).

The network of BW-significant protein-protein interactions (P-Pi) obtained in the STRING program is shown in Figure 3 and indicates a significant level of these interactions (*p* < 10^−16^). The basis of these P-Pi has been the pathways listed above according to the Gene Ontology resource. In addition, interestingly, there were founded common RNA expression patterns for two pair BW-causal genes such as *CDC25C* and *KIF20A* (RNA coexpression score 0.796; these genes were functionally (sQTL/eQTL) related to rs757647 *KDM3B*), *NKX2-1* and *SFTA3* (RNA coexpression score 0.473; these genes were functionally (sQTL/epigenetic modifications) related to rs999460 *NKX2-1*). P-Pis have been clustered into three groups (Figure 4D). The first group (Figure 4A) consists of 15 proteins (ADCY3, CDC25C, CENPO, DNAJC27, EFR3B, FTO, GPRC5B, IQCK, KIAA0368, KIF20A, etc.) associated with BMI-related phenotypes (fat body mass, obesity, height). The second group (Figure 4B) was formed by 22 proteins (ARL14EP, ARVCF, BSX, COMT, DNMT3A, ESR1, ESR2, FSHB, GHRH, GPR139, HACE1, INHBA, KISS1, LHCGR, NKX2-1, POMC, REN, SFTA3, TXNRD2, UGT2B4, ZBTB1, ZNF483) involved in hormone-dependent processes (regulation of hormone secretion, estrogen receptor activity, ovulation cycle process, female/male gonad development, etc.). The third group (Figure 4C) was formed by seven proteins (FAM53C, GC, KDM3B, LIN28B, MTHFD1, REEP2, SYNE2), the joint functional role of which is not clear.

## 4. Discussion

**Figure 5:**
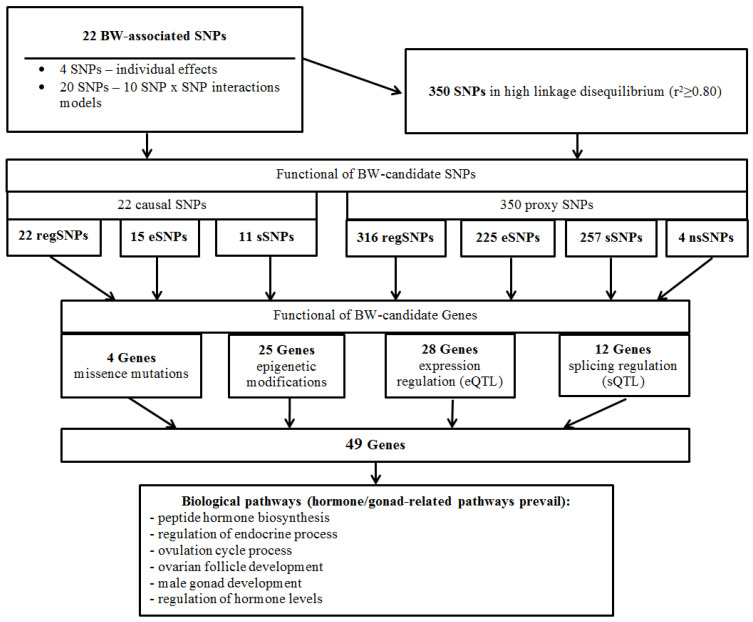
Study results outline.

This work has been the first study to report a link between maternal AAM-associated genes SNPs with offspring BW. Four loci independently (rs7589318 *POMC*; rs757647 *KDM3B*; rs1079866 *INHBA*; rs999460 *NKX2-1*) and 20 SNPs in interlocus interplays have been BW-correlated. BW-associated AAM-linked 22 SNPs and 350 proxy loci were functionally related to 49 genes relevant with pathways such as the hormone biosynthesis/process and female/male gonad development. The study results outline is presented in Figure 5.

In this work, it was found that the maternal allele T rs757647 *KDM3B* has the largest associations with increased offspring BW (β = 0.323). According to earlier genetic studies, the allele T rs757647 *KDM3B* has been linked with early AAM [22,80]; in addition, rs17171818 *KDM3B*, which linked to rs757647 *KDM3B* (r^2^ = 0.96), also was correlated with AAM [23]. Thus, the genetic factor of early maternal AAM was associated with a high offspring BW. Importantly, AAM is a key parameter of the woman (it reflects the state of the hypothalamic-pituitary-ovarian system) and is strongly correlated with woman’s fertility [17]. Early AAM is risk factor of various woman’s health-related disorders in adulthood such as impaired glucose metabolism/insulin resistance, obesity, metabolic syndrome, diabetes, cardiovascular diseases, etc. [16,21]. The limited literature on this topic also indicates the connection of early maternal AAM with an increased BMI of offspring [33,34,35]. Correlations of this orientation were found in the studies of Wang et al. among the Chinese population (a sample of 17,571 children showed a correlation between early maternal menarche and the offspring BMI of both boys and girls) [33]. In the works of Basso et al. in the USA population (with a sample of 31,474 black and white children was demonstrated that children of mothers with menarche <12 years were taller from age 1 and had higher BMI at ages 7–8 years) [34] and Ong et al. among the UK population (using a sample of 6009 children demonstrated an between association early AMM and characteristics of offspring such as higher height, weight, BMI, fat mass index at the age of 9 years) [35].

According to our data, SNP rs757647 *KDM3B* affects the interaction of DNA with the regulatory protein MAFK and TF CTCF, modulates gene expression due to localization in the enhancer regions of the *KDM3B* gene in amnion, placenta, ovary, adipose (mesenchymal stem cells, adipose nuclei, etc.); BW-raising allele T rs757647 *KDM3B* was correlated with low *REEP2* transcription in adipose (subcutaneous) and high *CDC25C* splicing in EBV-transformed lymphocytes. Additionally, rs757647 *KDM3B* has been high-linked with 18 sQTL SNPs in EBV-transformed lymphocytes (the splicing of two genes such as *FAM53C*,*CDC25C* was regulated by these polymorphisms).

The *KDM3B* gene encodes lysine-specific demethylase 3B, which demethylates mono- and dimethylated histone lysine 9 H3 (H3K9me1/H3K9me2) [81]. This modification of histones is quite important for the processes of stem cells proliferation/differentiation/migration [82]. Liu et al. demonstrated in a mouse model with *KDM3B* knockout that this gene is expressed in ovarian, fallopian tubes, uterus (endometrium/myometrium) [83]. In females with *KDM3B* knockout, decreased expression of *IGFBP-3* in the kidneys (by 53%) and a reduction in its content in the blood were observed, which led to accelerated degradation and a decrease in the IGF-1 blood concentration (by 36%). In addition, in the experiment, a decrease in the level of circulating 17beta-estradiol (>50%), irregular/prolonged estrous cycles, reduced ability to ovulate (by 45%) and fertilization (by 47%), decreased decidual response of the uterus (by 44%) were recorded [83]. The aforementioned data indicate the importance of *KDM3B* as a key H3K9 demethylase necessary to maintain circulating IGF-1 and 17beta-estradiol, the embryonic development processes, postnatal somatic growth and the functioning of the female reproductive system. It is assumed that *KDM3B*-mediated decrease in *IGFBP-3* expression, leading to rapid degradation of IGF-1, may also cause small body sizes [83].

Interestingly, the *CDC25C* gene (the level of splicing of this gene depends on polymorphism rs757647 *KDM3B* and 18 proxy loci) encodes a cell division cycle 25C protein involved in the cell division regulation by participating in cyclin B-related processes and overwhelming the p53 suppressive effects [84]. The FAM53 (family with sequence similarity 53 member C) protein encoded by the gene of the same name (*FAM53* splicing is controlled by SNP rs757647 *KDM3B* and 18 proxy variants), in interaction with transcription regulators, has a modifying effect on cell proliferation. There are GWAS data on the correlation of *CDC25C/FAM53C* genes polymorphisms with IGF-1 level, BMI, height [85,86,87].

The results of our work indicate the link between the maternal rs1079866 *INHBA* and offspring BW: the CC genotype was BW-raising (β = 0.110). Several previous genetic studies (GWAS, etc.) have showed association of rs1079866 *INHBA* with AAM [22,23,88], Tanner stage in girls [23], height adult [22,23,86], IGF-1 level [85], breast cancer risk [89]. Strongly coupled with rs1079866 *INHBA* SNP rs4141153 (r^2^ = 0.63) was also AAM-(GWAS)-linked [90,91]. Additionally, the BW-raising C allele has been associated with early menarche and low height adult [22,23,88]. SNP rs1079866 *INHBA* and LD SNP rs1079866 *INHBA* determine the DNA interaction manner with six TFs (e.g., HNF4,YY1, etc.) and were gene enhancer-related in adipose (mesenchymal stem cells; adipose nuclei; etc.). The *INHBA* gene encodes the βA subunit of the inhibin protein [84]. The inhibin βA subunit can also dimerize with the inhibin α and inhibin βB subunits to form inhibin A and activin AB, respectively. These hormones are involved in hypothalamic–pituitary–ovarian folliculogenesis and implantation processes. Inhibin A is synthesized by granulosa cells in the ovary and contributes to the suppression of gonadoliberin secretion in the hypothalamus and follicle-stimulating hormone in the adenohypophysis by the mechanism of negative feedback. *INHBA* is a modulator BW-significant TGF-β signaling pathway: overexpression of *INHBA* upregulated *TGF-β*-related genes (*TGF-β1/p-Smad2/p-Smad3/VEGF-A*), and respectively *INHBA* low expression downregulated activity of these genes [92]. Differential expression of the *INHBA* gene in the placenta has been shown [93], which may directly correlate with BW. Interesting experimental data on the potential mechanisms of the *INHBA* genes influence on the course of pregnancy (normal and complicated preeclampsia) were obtained in the work of Wang et al. [94]. The authors found that the *INHBA* gene together with the *OPRK1* and *TPBG* genes are key inflammation-related genes that affect the course of pregnancy (the expression of *INHBA* and *TPBG* genes in complicated preeclampsia pregnancy is higher, and the *OPRK1* gene is lower, compared with normal pregnancy). Wang et al. have shown that TFs such as SMARCC1, REL, ZNF274, RELB and RELA are involved in the regulation of the expression of *INHBA* (and *TPBG*), and the potential targets of the signaling pathways of these genes are microRNAs such as has-miR-1206 and has-miR-548k [94]. Increased expression of *INHBA* has been shown to contribute to human trophoblast invasion (via ALK3-BMPR2/ACVR2A-SMAD1/5/8-SMAD4 signaling pathways) whereby it has meaningful significance for early stages of embryonic implantation [95] and may have consequences for BW.

According to this work, maternal rs7589318 *POMC* was associated with offspring BW (β = 0.202). This locus affects DNA affinity to two TFs, ATF3 and CTCFL, and correlates with *RBJ* expression in peripheral blood. SNP rs7589318 *POMC* and proxy 11 variants were sQTL-impact (*ADCY3*) in adipose [subcutaneous] and thyroid, and eQTL-significant (*RBJ*) in blood. TF- and enhancer-involved locus, rs13010681 (this locus was located in enhancer position in amnion/placenta and determined DNA link with 10 TFs) and enhancer/promoter-related SNP in cultured trophoblast cells, cultured NK cells (CD56) of ectoderm and mesoderm, rs13022041, were strongly linked with rs7589318 *POMC*. Human NK cells in the pregnant uterus secrete growth-promoting factors, thereby having a positive effect on placental development, angiogenesis processes, which promotes fetal growth in the early stages of development [96,97]. In the previously presented materials rs7589318 *POMC* was AAM-associated [98]. Interestingly, several GWAS-significant for BMI-related polymorphisms’ traits are strongly linked to rs7589318 *POMC*, such as rs60925903 (r^2^ = 1.0; waist-to-hip ratio) [99], rs13032289 (r^2^ = 0.97; waist-to-hip ratio/waist-hip index) [100], rs2164808 (r^2^ = 0.64; body shape index) [100], rs6545975 (r^2^ = 0.36; BMI) [99], rs934778 (r^2^ = 0.27; weight) [101,102], rs13428823 (r^2^ = 0.20; height/waist circumference) [100,103] and rs13401333 (r^2^ = 0.20; BMI) [99]. The *POMC* gene encodes the inactive peptide proopiomelanocortin, which undergoes further cleavage by prohormonal convertases [84]. There are eight potential cleavage sites inside the *POMC*, which, depending on the tissue and the type of convertases, can lead to the formation of biologically active peptides involved in various physiological processes (immune modulation/energy metabolism/stimulation of melanocytes/pain suppression mechanisms, etc.). Basically, POMC is synthesized in the cells of the anterior pituitary gland, with the formation of a number of hormones: adrenocorticotropic hormone, β- and γ-lipotropic hormones, α-, β- and γ-melanocyto-stimulating hormones, β-endorphin [104]. Mutations/polymorphisms in the *POMC* gene are associated with early onset of obesity and adrenal insufficiency [105,106].

The *ADCY3* gene (this gene-splicing level in adipose [subcutaneous] and thyroid depends on BW-involved polymorphism rs7589318 *POMC* and 11 proxy loci) encodes membrane-linked enzyme, adenylyl cyclase 3, catalysing synthesis of cAMP (cyclic adenosine monophosphate) from ATP (adenosine triphosphate) in the process of signal transmission by the P-protein [107]. cAMP is believed to be involved in the intracellular signaling of molecules such as leptin, glucagon-like peptide 1, orexins, ghrelin and α-melanocyte-stimulating hormone [107,108]. It is a generally recognized scientific fact that the *ADCY3* gene/protein is involved in metabolic processes and obesity-significant pathways (data on this were obtained both on experimental models of *ADCY3*-knockout mice, Goto–Kakizaki rats and in numerous genetic associative studies in various human populations) [107,109,110,111]. Obese subjects are characterized by reduced *ADCY3* expression in adipose tissue [112]. According to our experimental/in silico data, BW-raising maternal allele A rs7589318 *POMC* was correlated with high *ADCY3* splicing level in adipose [subcutaneous] and thyroid. Importantly, *ADCY3* gene SNPs were GWAS-impact for BMI in children [113] and BMI across children aged 1 to 17 years [114].

In our study, maternal rs999460 *NKX2-1*’s link with the offspring BW (β = −0.176) was revealed. In previous studies, rs999460 *NKX2-1* polymorphism was associated with AAM [98], woman BMI [24] and endometrial hyperplasia [28]. The bioinformatic data obtained by us indicate the localization of this SNP in the promoter region of the *NKX2-1* gene in cultured NK cells (CD56) of the ectoderm, the effect on the affinity of DNA to five TFs (AIRE; Arid5a; Foxa; STAT; Pax-45) and the regulation of the *SFTA3* gene splicing in thyroid (BW-reducing allele A rs999460 *NKX2-1* was linked with high *SFTA3* splicing level). The *NKX2-1* gene encodes a transcription factor (thyroid transcription factor 1 (TTF1)), which binds and activates promoters of the thyroglobulin, thyroperoxidase and thyroid-stimulating hormone receptor genes [84]. In addition, this protein suppresses the expression of the transcription factor NR1D1, which, in turn, inhibits the transcription of genes involved in the metabolism of lipids and bile acids, adipogenesis, gluconeogenesis and inflammatory reactions of macrophages [115].

Interestingly, *NKX2-1* gene product—TTF1 is actively expressed during embryonic development in various organs (lungs, ventral forebrain, diencephalon, hypothalamus, etc.), including in the thyroid gland, which is crucial for both the early stages of thyroid development and embryonic development in general [116]. During the development of the thyroid gland, TTF1 together with other thyroid-significant TF (e.g., HHEX, FOXE1, PAX8) are jointly expressed both in thyroid progenitor cells and in thyroid mature follicular cells, ensuring its development, differentiation, homeostasis and function at the proper level [116]. It is important that in the absence of TTF1, the progenitor cells of the thyroid endure apoptosis and vanish at the embryonic development early stages, which leads to a decrease in thyroid follicular cells’ mass, its anomalous development and degradation [117]. Various abnormalities in the thyroid structure/functioning can lead to versatile endocrine-metabolic disorders in the neonatal period (e.g., congenital hypothyroidism) [Guan 2021], which directly correlate with BW (e.g., low-BW infants oftentimes demonstrate transient congenital hypothyroidism) [118]. Importantly, >50% of infants with low BW are diagnosed hypothyroxinemia at the thirtieth week of pregnancy [118].

In earlier genetic studies using the list of AAM-significant polymorphisms considered in this paper in the same population (Russian women of Central Russia), associations of a number of these polymorphic loci with AAM, BMI [24] and proliferative uterine diseases (endometriosis [29], endometrial hyperplasia (EH) [28], uterine fibroids (UL) [30]) were demonstrated. A comparative analysis of the results of this results and previously obtained data shows the link of BW-associated AAM-involved SNPs with AAM and BMI of women in this population (Supplementary Table S14): out of 22 BW-associated SNPs, eight loci were also BMI-associated (36.36%) and five loci were AAM-associated (22.73%); moreover, four loci such as rs4374421 *LHCGR*, rs4946651 *LIN28B*, rs1073768 *GHRH* and rs4633 *COMT* (18.18%) were associated with all three phenotypes under consideration (BW, AAM, BMI). Additionally, BW-associated AAM-involved SNPs were also involved in susceptibility to proliferative uterine diseases: out of 22 BW-associated SNPs, nine loci were also UL-associated (40.90%), 10 SNPs were EH-associated (45.45%) and eight loci were endometriosis-associated (36.36%); moreover, three loci such as rs4374421 *LHCGR*, rs12324955 *FTO* and rs1782507 *FSHB* (13.63%) were associated simultaneously with BW and the three proliferative diseases of the uterus indicated above (Supplementary Table S14). The aforementioned data indicate a significant similarity of the AAM-involved genetic determinants (polymorphisms) of the woman that determine the AAM, BMI and affect both the offspring BW and the development of the uterus proliferative diseases.

**Figure 6:**
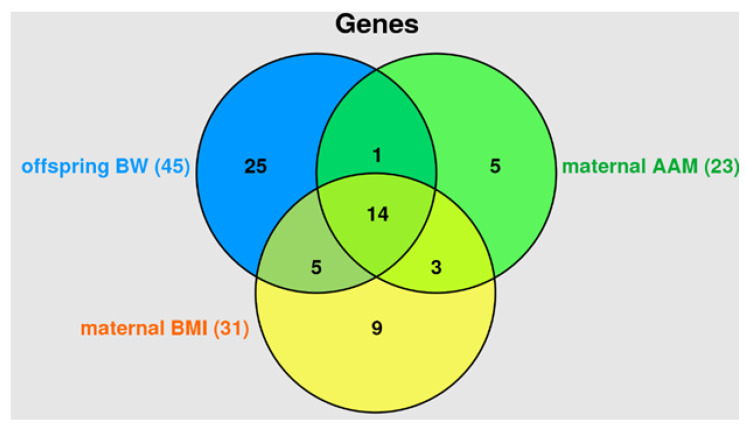
Venn diagram characterizing the syntropic effects of AAM-involved genes in offspring BW, maternal AAM and BMI (due to the functionality of BW-, AAM- and BMI-associated AAM-involved loci (22 SNPs [the data of this work], 13 and 14 SNPs [24] respectively) and proxy variants).

Among offspring BW-associated genes, 33.33% were maternal AAM-associated, 42.22% were maternal BMI-associated; while 31.11% (*GHRH*, *ADCY3*, *EFR3B*, *ARVCF*, *COMT*, *HACE1*, *LHCGR*, *MROH8*, *LIN28B*, *LIN28B-AS1*, *MANBAL*, *POMC*, *RBJ*, *TXNRD2*) were involved in the formation of all three phenotypes considered (offspring BW, maternal AAM and BMI) (Figure 6, genes functionally related to BW-,AAM-,BMI-associated loci (22 SNPs [the data of this work], 13 and 14 SNPs [24] respectively) and proxy SNPs) were considered). At the same time, 65.22% of maternal AAM-associated genes and 61.29% of maternal BMI-associated genes have been offspring BW-associated genes (Figure 4). Our data on the presence of common genetic factors offspring BW, maternal AAM and BMI correspond to modern literature materials [1,15]. Strong positive genetic correlations between BW and BMI/adult obesity [1] and causal genetic effect of BW on AAM (lower BW determined increased risk of earlier AAM) have been shown in several works [41,42]. The study by Tyrrell et al. convincingly showed that genetically raised maternal BMI was causally connected with increased offspring BW [15]. So, at the moment there is convincing genetic data on the association of higher pre-pubertal BMI (age 8 year) with earlier menarche [26,42], early menarche with high pubertal growth, pre-pubertal/adolescent/adult BMI [25,26,27,42], high maternal BMI with increased BW of the offspring [15]. At the same time, not high BW (as expected according to the above multi-stage correlation, such as high maternal BMI in childhood ─> early maternal menarche ─> high maternal BMI (adult) ─> high offspring BW will directly correlate with maternal AAM, but on the contrary, low BW is genetically associated with early menarche [41,42]. The above literature materials indicate the presence of common unidirectional genetic factors underlying the high maternal BMI and the high offspring BW and the ambiguous (including multidirectional) genetic correlations of maternal AAM and offspring BW.

Maternal AAM-related polymorphisms (rs7589318 *POMC*, rs757647 *KDM3B*, rs1079866 *INHBA*, rs999460 *NKX2-1*) associated with offspring BW according to this work can be recommended for use in practical medicine for the purpose of selection among women at the pre-pregnancy/early pregnancy stages at risk of birthing newborns with sufficiently high/low BW values and implementation among these women complex preventive/therapeutic measures aimed at preventing offspring with high/low BW. It should be noted that it is necessary to continue a more active study of the role of maternal AAM genes in the formation of offspring anthropometric characteristics in various ethnically different populations in order to establish “universal” genetic determinants of these phenotypic traits for the population as a whole, as well as studies on the influence of this group of maternal candidate genes on the course of pregnancy and its complications (placental insufficiency, fetal growth restriction, preeclampsia, etc.). The data established in this work can be the basis for subsequent research in this area as comparison indicators, which are obtained for the Europeans of Central Russia.

The limitation of this study is that these scientific results were obtained only for one population (Europeans of the Central region of Russia) and further studies (replicative) are needed to confirm the revealed facts of the connection of maternal AAM-related polymorphisms (rs7589318 *POMC*, rs757647 *KDM3B*, rs1079866 *INHBA*, rs999460 *NKX2-1*) with offspring BW in other European populations and among populations of other ethnic groups.

## 5. Conclusions

This study showed the dependence of the offspring BW on maternal AAM-associated gene polymorphisms that are functionally significant for hormone/gonad-related pathways. The data established in this work can be the basis for further research in this area and can also be used in the future in practical medicine.

## Figures and Tables

**Table 1:** Phenotypic characteristics of the study’s maternal and newborn participants.

Parameters	N (%)	Birth Weight, g $\bar{X}$ ± SD (min–max)	*p*-Value
Baseline characteristics			
Maternal age, years	716	26.56 ± 4.95 (16–45)	-
Maternal pre-pregnancy BMI, kg/m^2^	716	23.86 ± 4.32 (15.06–44.98)	-
Age at menarche, years	716	12.63 ± 1.06 (10–16)	
Birth weight, g	716	3142.96 ± 584.43 (1050–5220)	-
Gestational age, weeks	716	38.7 (24.0–41.0)	-
Infant gender, Male/Female	716	379 (52.93%)/337 (47.07%)	-
Maternal phenotypic characteristics and offspring birth weight			
Age, years			
16–25	75 (10.48)	3156.53 ± 519.23 (1490–4770)	0.22
21–25	238 (33.24)	3214.90 ± 551.87 (1140–5220)	
26–30	253 (35.34)	3102.98 ± 592.85 (1180–4440)	
>30	150 (20.95)	3089.47 ± 642.12 (1050–4340)	
Age at menarche, years			
early (<12)	64 (8.94)	3291.88 ± 510.58 (1490–3970)	**0.02**
average (12–14)	618 (86.31)	3131.51 ± 577.60 (1050–5220)	
late (>14)	34 (4.75)	3020.88 ± 783.82 (1420–4820)	
pre-pregnancy BMI, kg/m^2^			
underweight (<18.50)	43(6.01)	2855.12 ± 641.80 (1220–3990)	**<0.0001**
normal weight (18.50–24.99)	441 (61.59)	3109.62 ± 523.68 (1430–4510)	
overweight (25.00–29.99)	163 (22.77)	3304.36 ± 613.35(1050–4770)	
obesity (>30)	69 (9.22)	3354.20 ± 737.65 (1110–5220)	
The course of this pregnancy			
normal	283 (39.5)	3507.56 ± 325.80 (2510–4440)	**0.0001**
preeclampsia	168 (23.5)	3483.3 ± 399.99 (2630–5220)	
fetal growth restriction	191 (26.7)	2568.98 ± 311.79 (1050–2850)	
preeclampsia + fetal growth restriction	74 (10.3)	2457.43 ± 442.78 (1180–2970)	
Smoking:			
yes	436 (60.89)	3107.74 ± 586.01 (1050–4770)	0.07
no	280 (39.11)	3197.80 ± 578.75 (1110–5220)	
Alcohol:			
yes	550 (76.82)	3306.15 ± 509.36 (1420–4820	0.08
no	166 (23.18)	3539.52 ± 489.22 (2510–5220)	
History of arterial hypertension:			
yes	51 (7.12)	2780.00 ± 758.84 (1140–4820)	**0.0002**
no	665 (92.88)	3170.80 ± 559.95 (1050–5220)	
History of sexually transmitted diseases:			
yes	182 (25.42	3253.82 ± 526.15 (1430–4070)	0.10
no	534 (74.58)	3396.48 ± 509.78 (1420–5220)	
History of preeclampsia:			
yes	96 (13.41	3086.67 ± 534.93 (1530–4070)	0.34
no	620 (86.59)	3151.67 ± 591.65 (1050–5220))	
History of fetal growth restriction:			
yes	53 (7.40)	2488.21 ± 423.75 (1110–2970)	**<0.0001**
no	663 (92.60)	3195.30 ± 563.68 (1050–5220)	

*Note:* significant *p* values are shown in bold (the *p* indicator was calculated using the Kruskal–Wallis test).

**Table 2:** Associations of AAM-involved 49 SNPs with BW.

Minor Allele (SNP)	Gene	Chr	N	Model								
				Add			Dom			Rec		
				β	SE	P	β	SE	P	β	SE	P
T (rs1514175)	*TNNI3K*	1	714	0.023	0.035	0.497	0.044	0.050	0.377	0.009	0.066	0.898
T (rs466639)	*RXRG*	1	714	−0.002	0.050	0.968	0.007	0.057	0.909	−0.081	0.170	0.633
G (rs7538038)	*KISS1*	1	714	0.048	0.044	0.271	0.055	0.050	0.270	0.058	0.135	0.669
C (rs713586)	*RBJ*	2	712	−0.024	0.034	0.475	−0.023	0.051	0.652	−0.046	0.061	0.451
A (rs2164808)	*POMC*	2	714	0.034	0.035	0.335	0.049	0.054	0.362	0.038	0.059	0.518
A (rs7589318)	*POMC*	2	714	0.042	0.037	0.261	0.005	0.049	0.921	**0.202**	**0.085**	**0.015**
C (rs4374421)	*LHCGR*	2	707	−0.018	0.038	0.647	−0.029	0.049	0.559	−9.917	0.088	0.999
T (rs7579411)	*LHCGR*	2	710	−0.028	0.036	0.435	−0.022	0.053	0.681	−0.056	0.063	0.372
C (rs4953616)	*LHCGR*	2	713	0.012	0.039	0.760	−0.005	0.049	0.923	0.087	0.094	0.354
G (rs6732220)	*FSHR*	2	714	−0.018	0.040	0.662	−0.023	0.049	0.637	−0.014	0.104	0.895
G (rs4953655)	*FSHR*	2	714	0.002	0.040	0.954	−0.005	0.050	0.922	0.036	0.103	0.727
A (rs12617311)	*PLCL1*	2	711	0.036	0.036	0.329	0.035	0.050	0.480	0.071	0.075	0.346
C (rs6438424)	*IGSF11*	3	710	−0.043	0.035	0.215	−0.037	0.054	0.494	−0.083	0.060	0.168
A (rs2013573)	*UGT2B4*	4	714	0.016	0.046	0.732	0.020	0.051	0.694	−0.006	0.156	0.972
A (rs13111134)	*UGT2B4*	4	712	0.002	0.042	0.968	−0.020	0.049	0.691	0.134	0.122	0.272
C (rs222003)	*GC*	4	715	0.137	0.065	0.036	0.157	0.068	0.021	−0.238	0.377	0.529
C (rs222020)	*GC*	4	713	−0.008	0.056	0.881	−0.022	0.059	0.707	0.259	0.266	0.331
G (rs3756261)	*EGF*	4	713	−0.023	0.065	0.729	−0.027	0.068	0.687	0.087	0.379	0.819
T (rs757647)	*KDM3B*	5	709	0.037	0.041	0.366	−0.010	0.049	0.835	**0.323**	**0.110**	**0.004**
G (rs7766109)	*F13A1*	6	715	−0.019	0.035	0.580	0.006	0.055	0.919	−0.060	0.058	0.299
A (rs4946651)	*LIN28B*	6	714	0.050	0.035	0.158	0.074	0.052	0.156	0.053	0.064	0.412
C (rs7759938)	*LIN28B*	6	712	0.071	0.039	0.069	0.098	0.048	0.043	0.044	0.093	0.636
T (rs314280)	*LIN28B*	6	707	0.060	0.036	0.092	0.084	0.052	0.106	0.070	0.066	0.293
A (rs314276)	*LIN28B*	6	698	0.069	0.039	0.072	0.108	0.050	0.029	0.020	0.086	0.812
G (rs3020394)	*ESR1*	6	715	−0.071	0.037	0.053	−0.058	0.049	0.233	−0.182	0.080	0.023
G (rs1884051)	*ESR1*	6	715	−0.068	0.037	0.068	−0.065	0.049	0.182	−0.148	0.083	0.073
C (rs7753051)	*IGF2R*	6	714	−0.042	0.039	0.283	−0.074	0.049	0.130	0.031	0.093	0.740
C (rs1079866)	*INHBA*	7	714	**0.110**	**0.046**	**0.014**	0.119	0.052	0.022	0.189	0.155	0.223
T (rs2288696)	*FGFR1*	8	715	−0.043	0.044	0.331	−0.040	0.050	0.429	−0.128	0.144	0.377
A (rs10980926)	*ZNF483*	9	715	0.014	0.037	0.711	0.006	0.049	0.900	0.052	0.084	0.538
C (rs10441737)	*ZNF483*	9	703	0.023	0.038	0.534	0.016	0.049	0.747	0.071	0.084	0.398
C (rs10769908)	*STK33*	11	704	0.006	0.035	0.860	−0.038	0.055	0.493	0.061	0.059	0.301
G (rs555621)	*FSHB*	11	714	−0.037	0.035	0.297	−0.082	0.052	0.113	0.005	0.065	0.935
A (rs11031010)	*FSHB*	11	711	−0.018	0.051	0.729	−0.040	0.057	0.481	0.199	0.189	0.293
C (rs1782507)	*FSHB*	11	714	−0.024	0.036	0.510	0.006	0.049	0.904	−0.117	0.075	0.121
A (rs6589964)	*BSX*	11	715	0.031	0.035	0.368	0.055	0.054	0.312	0.026	0.059	0.663
A (rs1544410)	*VDR*	12	712	−0.013	0.036	0.725	−0.033	0.050	0.504	0.018	0.070	0.804
A (rs999460)	*NKX2−1*	14	714	−0.078	0.036	0.029	−0.068	0.049	0.167	**−0.176**	**0.073**	**0.014**
A (rs4986938)	*ESR2*	14	713	0.044	0.037	0.235	0.052	0.049	0.292	0.066	0.078	0.402
A (rs2241423)	*MAP2K5*	15	711	−0.014	0.045	0.760	−0.013	0.052	0.803	−0.036	0.133	0.785
T (rs12444979)	*GPRC5B*	16	714	−0.017	0.048	0.719	−0.020	0.055	0.714	−0.020	0.155	0.899
A (rs9939609)	*FTO*	16	715	0.046	0.034	0.181	0.075	0.052	0.153	0.043	0.060	0.472
A (rs12324955)	*FTO*	16	714	−4.004	0.038	0.999	−0.009	0.049	0.851	0.030	0.089	0.734
G (rs1398217)	*SKOR2*	18	710	−0.009	0.036	0.796	−0.045	0.052	0.393	0.041	0.066	0.539
G (rs2252673)	*INSR*	19	711	−0.036	0.043	0.409	−0.031	0.050	0.536	−0.110	0.127	0.386
A (rs1073768)	*GHRH*	20	713	0.017	0.035	0.634	0.015	0.055	0.785	0.031	0.060	0.609
C (rs4633)	*COMT*	22	714	0.015	0.034	0.660	−0.011	0.054	0.838	0.054	0.056	0.341
A (rs5930973)	*CD40LG*	23	704	−0.098	0.071	0.166						
T (rs3092921)	*CD40LG*	23	713	−0.002	0.066	0.970						

*Note:* Chr: chromosome; Add: additive; Dom: dominant; Rec: recessive; significant *p* values are shown in bold.

**Table 3:** Significant SNP × SNP interactions associated with BW.

N	SNP × SNP Interaction Models	NH	betaH	WH	NL	betaL	WL	P_perm_
Two-order interaction models (threshold level *p* < 4 × 10^−5^, real level *p* < 2.5 × 10^−5^)								
1	rs222003 *GC* × rs2013573 *UGT2B4*	1	0.726	17.98	0	-	-	<0.001
2	rs4946651 *LIN28B* × rs7538038 *KISS1*	2	0.353	18.46	1	−0.217	5.58	<0.001
3	rs7538038 *KISS1* × rs314280 *LIN28B*	2	0.364	19.57	1	−0.225	6.04	0.001
Three-order interaction models (threshold level *p* < 3 × 10^−6^, real level *p* < 5 × 10^−8^)								
1	rs222003 *GC* x rs4986938 *ESR2* × rs2013573 *UGT2B4*	2	1.259	32.19	3	−0.269	5.47	<0.001
2	rs555621 *FSHB* x rs7538038 *KISS1* × rs314280 *LIN28B*	5	0.474	30.41	1	−0.295	5.74	0.001
3	rs999460 *NKX2-1* x rs12444979 *GPRC5B* × rs4374421 *LHCGR*	3	0.527	31.90	2	−0.504	6.88	<0.001
Four-order interaction models (threshold level *p* < 2 × 10^−7^, real level *p* < 3 × 10^−13^)								
1	rs10441737 *ZNF483* × rs1073768 *GHRH* × rs7589318 *POMC* × rs4633 *COMT*	8	0.944	55.95	6	−0.495	23.01	<0.001
2	rs713586 *RBJ* × rs3020394 *ESR1* × rs6589964 *BSX* × rs12324955 *FTO*	5	0.551	13.57	11	−1.065	55.72	<0.001
3	rs713586 *RBJ* × rs3020394 *ESR1* × rs1782507 *FSHB* × rs12324955 *FTO*	4	0.465	12.05	10	−0.886	56.83	<0.001
4	rs713586 *RBJ* × rs6589964 *BSX* × rs12324955 *FTO* × rs1514175 *TNNI3K*	5	1.142	23.63	7	−1.299	55.55	<0.001

*Note:* The results were obtained using the MB-MDR method with adjustment for covariates; NH, number of significant high risk genotypes in the interaction; beta H, regression coefficient for high risk exposition in the step2 analysis; WH, Wald statistic for high risk category; NL, number of significant low risk genotypes in the interaction; beta L, regression coefficient for low risk exposition in the step2 analysis; WL, Wald statistic for low risk category; P_perm_, permutation *p*-value for the interaction model (1000 permutations).

## Data Availability

The data generated in the present study are available from the corresponding author upon reasonable request.

## Supplementary Materials

The following supporting information can be downloaded at: https://www.mdpi.com/article/10.3390/life13071525/s1. Table S1. The literature data about associations of the studied polymorphisms with age at menarche and some menarche-related traits. Table S2. The 50 SNPs analyzed in the present study (HaploReg (v.4.1)). Table S3. Distribution of genotypes of 49 SNPs in the study group. Table S4. Associations of haplotypes with BW. Table S5. Associations of genotype combinations with birth weight within the most significant models of gene-gene interactions. Table S6. The regulatory potential of the 22 BW-associated SNPs and the SNPs in high linkage disequilibrium (r2 > 0.80) (HaploReg (v4.1, update 05.11.2015) [54]. Table S7. Cis-eQTL values of the BW-associated SNPs in blood (according to Blood eQTL browser (http://genenetwork.nl/bloodeqtlbrowser/ accessed on 8 February 2023). FDR ≤ 0.05). Table S8. Cis-eQTL values of the SNPs in high LD (r2 ≥ 0.80) with the BW-associated SNPs in blood (according to Blood eQTL browser (http://genenetwork.nl/bloodeqtlbrowser/), FDR ≤ 0.05). Table S9. eQTL values of the BW-associated SNPs (according to Genotype-Tissue Expression (GTEx) (http://www.gtexportal.org/)) (*p* < 8 × 10^−5^, FDR ≤ 0.05). Table S10. eQTL values of the SNPs in high LD (r2 ≥ 0.80) with the BW-associated SNPs (according to Genotype-Tissue Expression (GTEx) (http://www.gtexportal.org/)) (*p* < 8 × 10^−5^, FDR ≤ 0.05). Table S11. sQTL values of the BW-associated SNPs (according to Genotype-Tissue Expression (GTEx) (http://www.gtexportal.org/)) (*p* < 8 × 10^−5^, FDR ≤ 0.05). Table S12. eQTL values of the SNPs in high LD (r2 ≥ 0.80) with the BW-associated SNPs (according to Genotype-Tissue Expression (GTEx) (http://www.gtexportal.org/)) (*p* < 8 × 10^−5^, FDR ≤ 0.05). Table S13. Gene set enrichment analysis of biological pathways with 49 BW-associated genes (BW-associated polymorphisms and SNPs in high LD (r2 ≥ 0.80) functionality in various tissue/organs) (Gene Ontology Portal tools, http://geneontology.org). Table S14. Association of BW-associated AMM-involved SNPs with maternal age at menarche and BMI in the studied population. References [22,23,24,39,80,88,98,103,110,119,120,121,122,123,124,125,126,127,128,129,130,131,132,133,134,135,136,137,138,139,140,141,142,143,144,145,146,147,148,149,150,151,152,153,154,155,156,157,158,159,160,161,162,163,164,165,166] are cited in the supplementary materials.
