# Supplementary material for: Maternal Age at Menarche Gene Polymorphisms Are Associated with Offspring Birth Weight
Source: Life (Basel). 2023 Jul 7;13(7):1525. doi: 10.3390/life13071525 (PMC10381708; doi:10.3390/life13071525)
Supplement: Supplementary file 1 [file life-13-01525-s001.zip › Supplementary Table S1.pdf]

Supplementary Table S1. The literature data about associations of the studied polymorphisms with age at menarche and some menarche-related traits.

Chr	SNP	Gene/Region	Phenotype	Association (significance) (associated allele/genotype)	Reference
1	rs1514175	*TNNI3K*	Age at menarche	−12.2 d (p=4.0 × 10^{-7}) (A)	[133]
			**Body mass index**	**0.07 kg/m^2 (p=8 x10^{-14}) (A)**	**[110]**
			**Body mass index**	**0.06 unit (p=3 x10^{-11}) (A)**	**[136]**
			Body mass index	0.20 kg/m^2 (p=7.7× 10^{-4}) (A)	[138]
1	rs466639	*RXRG*	**Age at menarche**	**-4.2 w (p= 1 x10^{-13}) (T)**	**[22]**
			Body mass index	increasing (p= 0.011) (T)	[22]
			Age at menarche	-4.1 w (p= 0.023) (T)	[80]
			**Age at menarche**	**0.08 unit (p= 2 x10^{-24}) (C)**	**[23]**
1	rs7538038	*KISS1*	Age at menarche	-0.061 y (p_{adj}=0.012) (G)	[98]
2	rs713586	*RBJ*	Age at menarche	11.7 d (p=8.4 × 10^{-7}) (T)	[133]
			**Body mass index**	**0.14 kg/m^2 (p= 6 x10^{-22}) (C)**	**[110]**
2	rs2164808	*POMC*	Age at menarche	0.046 y (p_{adj}=0.038) (T)	[98]
2	rs7589318	*POMC*	Age at menarche	0.048 y (p_{adj}=0.047)(A)	[98]
2	rs4374421	*LHCGR*	Age at menarche	0.060 y (p_{adj}=0.017) (C)	[98]
2	rs7579411	*LHCGR*	Age at menarche	0.058 y (p_{adj}=0.013)(T)	[98]
2	rs4953616	*LHCGR*	Age at menarche	-0.065 y (p_{adj}=0.006) (T)	[98]
2	rs6732220	*FSHR*	Age at menarche<br>Age at menarche	0.17 y (p=0.0018,p_{bonf}>0.05)(G) *<br>0.34 y (p= 3 x10^{-5}) epistasis	[150]
2	rs4953655	*FSHR*	Age at menarche<br>Age at menarche	0.17 y (p=0.0023,p_{bonf}>0.05)(G) *<br>0.34 y (p= 3 x10^{-5}) epistasis	[150]
2	rs12617311	*PLCL1*	**Age at menarche**	**-3.0 w (p= 6 x10^{-13}) (A)**	**[22]**
			Age at menarche	-4.7 w (p=0.004) (A)	[80]
3	rs6438424	*IGSF11*	**Age at menarche**	**-2.7 w (p= 1 x10^{-13}) (A)**	**[22]**
			Height adult	-0.013 Zscore (p=0.0004) (A)	[22]
			Age at menarche	-3.7 w (p=0.008) (A))	[80]
			Age at menarche	0.17 y (p_{perm}=0.005) (A))	[24]
4	rs2013573	*UGT2B4*	Age at menarche	0.060 y (p_{adj}=0.019) (C)	[98]
4	rs13111134	*UGT2B4*	Age at menarche	0.055 y (p_{adj}=0.042) (G)	[98]
			Age at menarche	0.431y (p_{bonf}=0.044) (A)	[165]
4	rs222003	*GC*	Plasma levels of 25(OH)D and 1,25$(OH)_2$D	(p>0.05)	[132]
			Compression strength index (male)	(p>0.05)	[163]
4	rs222020	*GC*	Plasma 25(OH)D concentrations	(p=0.004)	[166]
			Compression strength index (male)	(p=0.0042)	[163]
			Adolescent idiopathic scoliosis	(p<0.05)	[159]
4	rs3756261	*EGF*	Birth weight	lower (p<0.0001) (A)<br>(4 snps haplotype)	[122]
5	rs757647	*KDM3B*	**Age at menarche**	**-2.4w(p=5.4x10^{-8},p_{bonf}>0.05)(A)***	**[22]**
			Age at menarche	-4.2 w (p=0.003) (A)	[80]
6	rs7766109	*F13A1*	Body mass index,<br>pressure insulin response,<br>triglycerides,<br>high density lipoprotein cholesterol in PCOS	(p=0.013)<br>(p=0.015)<br>(p=0.001)<br>(p=0.012)	[153]
6	rs4946651	*LIN28B*	**Age at menarche**	**0.085 y (p= 3 x10^{-8}) (A)**	**[137]**
6	rs7759938	*LIN28B*	**Age at menarche**	**0.09 y (p= 7 x10^{-9}) (C)**	**[147]**
			**Age at menarche**	**6.4 w (p= 5 x10^{-60}) (C)**	**[22]**
			Height adult	-0.042 Zscore (p=8.7 x10^{-18}) (T)	[22]
			Age at menarche	8.3 w (p= 4 x10^{-6}) (C)	[80]
			**Height**	**-0.045 unit (p= 8 x10^{-31}) (T)**	**[143]**
			**Late pubertal growth**	**0.11 unit (p= 4 x10^{-9}) (C)**	**[125]**
			**Age at menarche**	**0.12 unit (p= 8 x10^{-110}) (C)**	**[23]**
			Tanner stage	-0.080 (p=3.42 x10^{-8}) (C)	[23]
			Tanner stage (boys)	-0.063 (p=0.012) (C)	[23]
			Tanner stage (girls)	-0.088 (p=5.57 x10^{-7}) (C)	[23]
			Height adult	increasing (p=8.69 x10^{-18}) (C)	[23]
			Height adult	0.042 cm (p= 2 x10^{-6}) (C)	[144]
			Pubertal growth	(p=5x10^{-11})	[161]
			Age at menarche in PCOS	earlier (p=0.006) (T)	[120]

6	rs314280	*LIN28B*	**Age at menarche**	**0.086 y (p= 2 x10^{-8}) (T)**	**[137]**
			**Age at menarche**	**1.2 m (p=2 x10^{-14}) (T)**	**[156]**
			Age at menarche (EA)	0.08 y (p=2 x10^{-8}) (T)	[121]
			Age at menarche (Asians)	0.13 y (p= 0.0012) (T)	[121]
6	rs314276	*LIN28B*	**Age at menarche**	**- 0.14 y (p= 4 x10^{-16}) (C)**	**[39]**
			Height	-0.37 cm (p=3.6 x10^{-7}) (C)	[39]
			Breast development in girls	earlier (p = 0.001)	
			Voice breaking	earlier (p = 0.006)	
			Height growth in girls	faster tempo (p= 0.00008)	
			**Puberty onset**	**0.08 unit (p= 2 x10^{-8}) (C)**	**[126]**
6	rs3020394	*ESR1*	Age at menarche	-0.12 y (p=0.0081,p_{bonf}>0.05)(A)*	[150]
			Hip fractures	OR=1.66 (p=0.0004)	[160]
6	rs1884051	*ESR1*	Age at menarche	-0.13 y (p=0.0035,p_{bonf}>0.05)(A)*	[150]
			Hip fractures	OR=1.46 (p=0.0004)	[160]
			Body weight,	lower (p = 0.007) (A),	[129]
			body mass index ,	(p = 0.003) (A) ,	
			waist-hip ratio,	(p= 0.011) (A) ,	
			fat body mass,	(p= 0.010) (A),	
			body fat percentage	(p= 0.040) (A),	
			obesity in men	decreased (p=0.04) (A)	
			Type 2 diabetes	(p=0.011)	[127]
6	rs7753051	*IGF2R*	Age at menarche	0.14 y (p=0.001, p_{bonf}>0.05) (T) *	[150]
			Age at menarche	0.34 y (p= 3 x10^{-5}) epistasis	[150]
			Age at menarche	0.500 y (p=0.046,p_{bonf}>0.05)(A)*	[165]
7	rs1079866	*INHBA*	**Age at menarche**	**3.9 w (p=6 x10^{-14}) (G)**	**[22]**
			Height adult	-0.016 Zscore (p=0.015) (C)	[22]
			**Age at menarche**	**0.07 unit (p=9 x10^{-24}) (G)**	**[23]**
			Tanner stage	-0.054 (p=0.005) (G)	[23]
			Tanner stage (girls)	-0.058 (p=0.015) (G)	[23]
			Height adult	increasing (p=0.015) (G)	[23]
			Age at menarche	0.13 y (p=0.037) (G)	[88]
8	rs2288696	*FGFR1*	Age at menarche	-0.058 y (p_{adj}=0.033) (G)	[98]
9	rs10980926	*ZNF483*	**Age at menarche**	**2.5 w (p= 4 x10^{-11}) (A)**	**[22]**
			Height adult	-0.015 Zscore (p=0.0017) (G)	[22]
			Age at menarche	2.6 w (p= 0.047) (A)	[80]
9	rs10441737	*ZNF483*	**Age at menarche (EA)**	**-2.78 unit (p= 4 x10^{-15}) (T)**	**[128]**
11	rs10769908	*STK33*	Age at menarche	9.6 d (p=7.8× 10^{-5}) (T)	[133]
11	rs555621	*FSHB*	Age at menarche	0.059 y (p_{adj}=0.0011) (C)	[98]
11	rs11031010	*FSHB*	Age at menarche	0.079 y (p_{adj}=0.0037) (A)	[98]
			PCOS	(p=4.27 × 10^{-8})	[157]
11	rs1782507	*FSHB*	Age at menarche	0.046 y (p_{adj}=0.021) (T)	[98]
11	rs6589964	*BSX*	**Age at menarche**	**-2.7 w (p= 2 x10^{-12}) (A)**	**[22]**
			Height adult	-0.014 Zscore (p=0.0025) (A)	[22]
12	rs1544410	*VDR*	Vitamin D deficiency in children	OR=2.33 (p=0.041) (G)	[141]
			Vitamin D vs testosterone level, free androgen index in women	(p<0.05)	[143]
			23% of the variation in serum 25-hydroxyvitamin D concentrations in children	(p<0.05)	[123]
			Testosterone level and free androgen index in women	p<0.002	[142]
			Osteoporosis	OR=0.61-0.70 (p<0.05)	[140]
			Bone mineral density at the lumbar spine in girls with adolescent idiopathic scoliosis	lower levels (p<0.05) (TT)	[155]
			Adolescent idiopathic scoliosis	(p<0.05)	[159]
14	rs999460	*NKX2-1*	Age at menarche	0.046 y (p_{adj}=0.039) (T)	[98]
14	rs4986938	*ESR2*	Age at menarche	-7.0 m (p=0.005) (G)	[154]
15	rs2241423	*MAP2K5*	Age at menarche	13.1 d (p=6.1× 10^{-6}) (A)	[133]
			**Body mass index**	**0.13 kg/m^2 (p= 1 x10^{-18}) (G)**	**[110]**
			Obesity	OR = 0.79 (p= 0.029) (A)	[152]
			Body mass index	−0.092 unit (p = 0.028) (A)	[152]
			Children obesity	(p<0.005)	[145]
			Obesity	OR=1.34 (0.001) (G)	[159]
			Adulthood body mass index	(p<0.05)	[146]

			Body mass index	0.11 kg/m^2 (p=0.028) (G)	[138]
16	rs12444979	*GPRC5B*	Age at menarche	13.5 d (p=2.6× 10^{-4}) (T)	[133]
			**Body mass index**	**0.17 kg/m^2 (p=3 x10^{-21}) (C)**	**[110]**
			Body mass index	(p<0.05)	[119]
			Body mass index (EA)	(p=0.014)	[164]
			Body fat mass (EA)	(p=0.002)	[164]
16	rs9939609	*FTO*	**Age at menarche**	**-2.1 w (p= 3 x10^{-8}) (A)**	**[22]**
			Body mass index	increasing (p= 6.3 x10^{-17}) (A)	[22]
			**Body mass index**	**0.33 kg/m^2 (p= 4 x10^{-51}) (A)**	**[162]**
			**Body mass index**	**0.36 kg/m^2 (p= 3 x10^{-35}) (A)**	**[134]**
			**Obesity**	**OR=1.25 (p= 1 x10^{-20}) (A)**	**[148]**
			Obesity	OR=9.86 (p=0.026) (AA)	[139]
			Obesity	OR=2.03 (p=0.002) (AA)	[131]
			Obesity	OR=1.72 (p=0.009) (AA)	[149]
			Obesity	OR = 1.39 (p < 0.01) (A)	[151]
			Body mass index	increasing (p=0.0059) (A)	[158]
			Extreme obesity	OR=1.46 (p=1.42 x10^{-11}) (A)	[124]
16	rs12324955	*FTO*	Age at menarche	-0.15 y (p=0.0057,p_{bonf}>0.05)(A)*	[150]
18	rs1398217	*SKOR2*	**Age at menarche**	**-2.7 w (p= 2 x10^{-13}) (G)**	**[22]**
19	rs2252673	*INSR*	PCOS	OR=1.32 (p=0.006) (G)	[135]
			PCOS	(p<0.05)	[130]
20	rs1073768	*GHRH*	Age at menarche	-0.046 y (p_{adj}=0.045) (C)	[98]
22	rs4633	*COMT*	Age at menarche	0.13 y (p=0.0097,p_{bonf}>0.05) (T) *	[150]
X	rs5930973	*CD40LG*	Age at menarche	0.101 y (p_{adj}=0.0067) (A)	[98]
X	rs3092921	*CD40LG*	Age at menarche	0.074 y (p_{adj}=0.037) (C)	[98]

Note: The data from the GWAS are highlighted in bold. * putative candidate loci age at menarche (crude p<0.05, corrected for multiple comparisons p_{bonf}>0.05).
