# Supplementary material for: Maternal Age at Menarche Gene Polymorphisms Are Associated with Offspring Birth Weight
Source: Life (Basel). 2023 Jul 7;13(7):1525. doi: 10.3390/life13071525 (PMC10381708; doi:10.3390/life13071525)
Supplement: Supplementary file 1 [file life-13-01525-s001.zip › Supplementary Table S14.pdf]

**Supplementary Table S14.** Association of BW-associated AMM-involved SNPs with maternal age at menarche and BMI in the studied population*.

| Chr | SNP | Gene | offspring BW | Maternal phenotypes | | | | |
|---|---|---|---|---|---|---|---|---|
| | | | | biological | | disorder | | |
| (1) | (2) | (3) | (4) | age at menarche* (5) | BMI* (6) | uterine leiomyoma (7)** | endometrial hyperplasia (8)*** | endometriosis (9)**** |
| **1** | **rs1514175** | ***TNNI3K*** | yes | | | yes | | yes |
| 1 | rs466639 | *RXRG* | | | | | | |
| 1 | rs7538038 | *KISS1* | yes | | | | yes | |
| 2 | rs713586 | *RBJ* | yes | | | yes | | yes |
| 2 | rs2164808 | *POMC* | | yes | yes | | | |
| 2 | rs7589318 | *POMC* | yes | | | yes | | |
| 2 | rs4374421 | *LHCGR* | yes | yes | yes | yes | yes | yes |
| 2 | rs7579411 | *LHCGR* | | yes | | yes | | yes |
| 2 | rs4953616 | *LHCGR* | | | yes | yes | | |
| 2 | rs6732220 | *FSHR* | | | | | yes | |
| 2 | rs4953655 | *FSHR* | | | yes | yes | yes | |
| 2 | rs12617311 | *PLCL1* | | yes | yes | | | |
| 3 | rs6438424 | *3q13.32* | | yes | | | yes | yes |
| 4 | rs2013573 | *UGT2B4* | yes | | | yes | yes | |
| 4 | rs13111134 | *UGT2B4* | | | | | yes | |
| 4 | rs222003 | *GC* | yes | | | | | |
| 4 | rs222020 | *GC* | | | | yes | | |
| 4 | rs3756261 | *EGF* | | | | | | |
| 5 | rs757647 | *KDM3B* | yes | | | | | |
| 6 | rs7766109 | *F13A1* | | | | | yes | |
| 6 | rs4946651 | *LIN28B* | yes | yes | yes | yes | | |
| 6 | rs7759938 | *LIN28B* | | yes | | | yes | yes |
| 6 | rs314280 | *LIN28B* | yes | | yes | | | |
| 6 | rs314276 | *LIN28B* | | yes | | yes | yes | |
| 6 | rs3020394 | *ESR1* | yes | | yes | | | |
| 6 | rs1884051 | *ESR1* | | | | | | |
| 6 | rs7753051 | *IGF2R* | | | | yes | | |
| 7 | rs1079866 | *INHBA* | yes | | | | | |
| 8 | rs2288696 | *FGFR1* | | | | | | |
| 9 | rs10980926 | *ZNF483* | | | | yes | yes | yes |
| 9 | rs10441737 | *ZNF483* | yes | | | | yes | yes |
| 11 | rs10769908 | *STK33* | | yes | | yes | | |
| 11 | rs555621 | *FSHB* | yes | | yes | yes | yes | yes |
| 11 | rs11031010 | *FSHB* | | | | yes | yes | yes |
| 11 | rs1782507 | *FSHB* | yes | | | yes | yes | yes |
| 11 | rs6589964 | *BSX* | yes | yes | | | yes | yes |
| 12 | rs1544410 | *VDR* | | | yes | yes | | yes |
| 14 | rs999460 | *NKX2-1* | yes | | yes | | yes | |
| 14 | rs4986938 | *ESR2* | yes | | | | yes | |
| 15 | rs2241423 | *MAP2K5* | | | | yes | | |
| 16 | rs12444979 | *GPRC5B* | yes | | | | | |
| 16 | rs9939609 | *FTO* | | | | | | |
| 16 | rs12324955 | *FTO* | yes | | | yes | yes | yes |
| 18 | rs1398217 | *SKOR2* | | | yes | | | |
| 19 | rs2252673 | *INSR* | | yes | | | | |
| 20 | rs1073768 | *GHRH* | yes | yes | yes | | | yes |
| 22 | rs4633 | *COMT* | yes | yes | yes | yes | yes | |
| 23 | rs5930973 | *CD40LG* | | | | | | |
| 23 | rs3092921 | *CD40LG* | | | | | | |

*Note:* **yes**: associated.

BW-associated SNP associated with one of the three maternal phenotype (uterine leiomyoma / endometrial hyperplasia / endometriosis).

BW-associated SNP associated with two / three of the three maternal phenotypes (uterine leiomyoma / endometrial hyperplasia / endometriosis).

BW-associated SNP associated with one of the two maternal phenotype (age at menarche / BMI).

BW-associated SNP associated with two of the two maternal phenotype (age at menarche / BMI).

* [24]. ** [30]. *** [28]. **** [29].
