# Supplementary material for: Maternal Age at Menarche Gene Polymorphisms Are Associated with Offspring Birth Weight
Source: Life (Basel). 2023 Jul 7;13(7):1525. doi: 10.3390/life13071525 (PMC10381708; doi:10.3390/life13071525)
Supplement: Supplementary file 1 [file life-13-01525-s001.zip › Supplementary Table S3.pdf]

Supplementary Table S3. Distribution of genotypes of 49 SNPs in the study group.

Minor allele (rs)	Gene	Chr	Genotypes	Minor allele frequency	H_o	H_e	P
T (rs1514175)	*TNNI3K*	1	115/325/274	0.3887	0.455	0.475	0.270
T (rs466639)	*RXRG*	1	15/156/543	0.1303	0.219	0.227	0.323
G (rs7538038)	*KISS1*	1	24/251/439	0.2094	0.352	0.331	0.114
C (rs713586)	*RBJ*	2	140/332/240	0.4298	0.466	0.490	0.194
A (rs2164808)	*POMC*	2	153/363/198	0.4685	0.508	0.498	0.600
A (rs7589318)	*POMC*	2	65/300/349	0.3011	0.420	0.421	1.000
C (rs4374421)	*LHCGR*	2	59/312/336	0.3041	0.441	0.423	0.287
T (rs7579411)	*LHCGR*	2	132/369/209	0.4458	0.520	0.494	0.196
C (rs4953616)	*LHCGR*	2	51/304/358	0.2847	0.426	0.407	0.233
G (rs6732220)	*FSHR*	2	42/269/403	0.2472	0.377	0.372	0.841
G (rs4953655)	*FSHR*	2	43/251/420	0.2360	0.352	0.361	0.533
A (rs12617311)	*PLCL1*	2	85/319/307	0.3439	0.449	0.451	0.868
C (rs6438424)	*IGSF11*	3	150/351/209	0.4585	0.494	0.497	0.940
A (rs2013573)	*UGT2B4*	4	18/234/462	0.1891	0.328	0.307	0.086
A (rs13111134)	*UGT2B4*	4	30/272/410	0.2331	0.382	0.358	0.075
C (rs222003)	*GC*	4	3/105/607	0.0776	0.147	0.143	0.792
C (rs222020)	*GC*	4	6/146/561	0.1108	0.205	0.197	0.444
G (rs3756261)	*EGF*	4	3/104/606	0.0771	0.146	0.142	0.791
T (rs757647)	*KDM3B*	5	36/269/404	0.2405	0.379	0.365	0.355
G (rs7766109)	*F13A1*	6	162/359/194	0.4776	0.502	0.499	0.940
A (rs4946651)	*LIN28B*	6	124/354/236	0.4216	0.496	0.488	0.701
C (rs7759938)	*LIN28B*	6	52/318/342	0.2963	0.447	0.417	0.072
T (rs314280)	*LIN28B*	6	114/356/237	0.4130	0.504	0.485	0.352
A (rs314276)	*LIN28B*	6	63/321/314	0.3202	0.460	0.435	0.164
G (rs3020394)	*ESR1*	6	73/299/343	0.3112	0.418	0.429	0.541
G (rs1884051)	*ESR1*	6	68/301/346	0.3056	0.421	0.424	0.860
C (rs7753051)	*IGF2R*	6	54/330/330	0.3067	0.462	0.425	0.022
C (rs1079866)	*INHBA*	7	18/216/480	0.1765	0.303	0.291	0.305
T (rs2288696)	*FGFR1*	8	21/247/447	0.2021	0.346	0.323	0.063
A (rs10980926)	*ZNF483*	9	67/302/346	0.3049	0.422	0.424	0.930
C (rs10441737)	*ZNF483*	9	66/297/340	0.3051	0.423	0.424	0.929
C (rs10769908)	*STK33*	11	155/359/190	0.4751	0.510	0.499	0.597
G (rs555621)	*FSHB*	11	117/359/238	0.4153	0.503	0.486	0.397
A (rs11031010)	*FSHB*	11	12/156/543	0.1266	0.219	0.221	0.865
C (rs1782507)	*FSHB*	11	84/332/298	0.3501	0.465	0.455	0.622
A (rs6589964)	*BSX*	11	156/359/200	0.4692	0.502	0.498	0.881
A (rs1544410)	*VDR*	12	99/334/279	0.3736	0.469	0.468	1.000
A (rs999460)	*NKX2-1*	14	90/321/303	0.3508	0.450	0.456	0.743
A (rs4986938)	*ESR2*	14	77/328/308	0.3380	0.460	0.448	0.503
A (rs2241423)	*MAP2K5*	15	25/206/480	0.1800	0.290	0.295	0.612
T (rs12444979)	*GPRC5B*	16	18/178/518	0.1499	0.249	0.255	0.557
A (rs9939609)	*FTO*	16	146/344/225	0.4448	0.481	0.494	0.496
A (rs12324955)	*FTO*	16	59/295/360	0.2892	0.413	0.411	0.928
G (rs1398217)	*SKOR2*	18	116/361/233	0.4176	0.509	0.486	0.248
G (rs2252673)	*INSR*	19	27/240/444	0.2068	0.338	0.328	0.493
A (rs1073768)	*GHRH*	20	149/371/193	0.4691	0.520	0.498	0.259
C (rs4633)	*COMT*	22	176/339/199	0.4839	0.475	0.500	0.203
A (rs5930973)	*CD40LG*	23	5/75/624	0.0604	0.107	0.113	0.101
T (rs3092921)	*CD40LG*	23	3/104/606	0.0771	0.146	0.142	0.791

Note: Chr – chromosome; H_o – observed heterozygosity; H_e – expected heterozygosity; P – level of significance.
