# Supplementary material for: Maternal Age at Menarche Gene Polymorphisms Are Associated with Offspring Birth Weight
Source: Life (Basel). 2023 Jul 7;13(7):1525. doi: 10.3390/life13071525 (PMC10381708; doi:10.3390/life13071525)
Supplement: Supplementary file 1 [file life-13-01525-s001.zip › Supplementary Table S4.pdf]

Supplementary Table S4. Associations of haplotypes with BW.

Haploblocks (gene) and SNPs	Haplotypes	Frequency	β	P
H1 (*FSHB*) rs555621-rs11031010-rs1782507	ACC	0.351	-0.027	0.466
	GAA	0.126	-0.017	0.739
	GCA	0.289	-0.034	0.380
	ACA	0.234	0.080	0.047
H2 (*ZNF483*) rs1098092-rs10441737	AC	0.304	0.023	0.537
	GT	0.693	-0.017	0.655
H3 (ESR1) rs3020394-rs1884051	GG	0.301	-0.074	0.046
	AA	0.685	0.064	0.078
H4 (*LIN28B*) rs4946651-rs7759938	AC	0.296	0.071	0.069
	AT	0.124	-0.010	0.840
	GT	0.58	-0.053	0.133
H5 (*UGT2B4*) rs2013573-rs13111134	AA	0.188	0.012	0.801
	GA	0.044	-0.028	0.739
	GG	0.768	-0.003	0.946
H6 (*FSHR*) rs6732220-rs4953655	GG	0.235	-0.001	0.973
	GA	0.012	-0.252	0.116
	CA	0.752	0.013	0.739
H7 (*LHCGR*) rs7579411-rs4953616	TC	0.279	0.014	0.727
	TT	0.166	-0.073	0.122
	CT	0.551	0.027	0.450
H8 (*POMC*) rs2164808-rs7589318	AA	0.301	0.041	0.270
	AG	0.168	-0.004	0.932
	GG	0.532	-0.034	0.336
